# Association between changes in depressive symptoms and hip fracture among middle-aged and older Chinese individuals: a prospective cohort study

**DOI:** 10.1186/s12877-022-03484-8

**Published:** 2022-11-08

**Authors:** Zhiwei Lian, Chunsu Zhu, Haowen Yuan, Jianmin Wang

**Affiliations:** 1 Clinical Oncology School of Fujian Medical University, Fujian Cancer Hospital, Fuzhou, China; 2 Innovation Center for Cancer Research, Clinical Oncology School of Fujian Medical University, Fujian Cancer Hospital, No. 420, Fuma Road, Jinan District, 350014 Fuzhou, China; 3 Fujian Key Laboratory of Advanced Technology for Cancer Screening and Early Diagnosis, Fujian Cancer Hospital, Fuzhou, China; 4 School of Public Health, Peking University, Beijing, China

**Keywords:** Hip fracture, Depressive symptoms, Epidemiology, Longitudinal cohort study

## Abstract

**Background:**

Although studies have shown that depressive symptoms are associated with an increased risk of hip fracture (HF). Depressive symptoms are dynamic, and it is unclear whether HF risk persists if depressive symptoms remit. This study aims to examine the associations between changes in depressive symptoms and HF risk.

**Methods:**

Data were from the China Health and Retirement Longitudinal Study from 2011 to 2018. Depressive symptoms were measured using the 10-item version of the Center for Epidemiological Studied Depression scale (cutoff ≥ 10). Changes in depressive symptoms were classified into four groups by two successive surveys (stable low/no, recent-onset, recently remitted, and stable high depressive symptoms). Multivariable logistic regressions were performed to assess whether changes in depressive symptoms were associated with HF incidents reported through 2018, adjusting for age, sex, educational level, marital status and other potential confounding factors.

**Results:**

In total, 8574 participants were included, 265 (3.1%) of whom had reported HF incidents in the subsequent 5-year period. Participants with recent-onset (OR = 1.97, 95% CI = 1.40–2.77) or stable high (OR = 2.15, 95% CI = 1.53–3.02) symptoms had a higher risk of HF than those with stable low/no depressive symptoms, whereas those with improved depressive symptoms (OR = 1.27, 95% CI = 0.89–1.82) had no elevation in HF risk.

**Conclusion:**

Stable high and recent-onset depressive symptoms were associated with increased HF risk, and no elevated HF risk was observed if symptoms remitted, suggesting that strategies to reduce depressive symptoms may be beneficial for HF prevention.

**Supplementary Information:**

The online version contains supplementary material available at 10.1186/s12877-022-03484-8.

## Introduction

Hip fracture (HF) is a major public health issue both worldwide and in China and is associated with an increased mortality rate, high disability rate and considerable medical expenditure[1, 2]. It was reported that 20-30% of patients would die in the first year after an HF event[3, 4], 50% of those who survived would lose functional independence, and approximately 30% would eventually become fully dependent[5]. Moreover, approximately 4.5 million people are disabled from HF each year, and the number is expected to increase to 21 million in the next four decades[6]. In addition, available data suggest that HF is associated with high medical costs through several aspects, including hospitalization, rehabilitation, nursing home placement and other costs[7]. With the aging of the global population, the absolute number of HFs in the world is predicted to increase from 1.26 million in 1990 to 4.5 million by 2050, more than 50% of which will occur in Asia, particularly in China[8–10]. China houses nearly one-fifth of the world’s population and is experiencing rapid population aging, with 164.5 million citizens aged 65 and above in 2019, and the number is expected to be 365 million by 2050[11]. A national study in China showed that between 2012 and 2016, the total absolute number of HFs among adults aged 55 years and older increased approximately 4-fold, and the related medical costs for hospitalization also rose steeply from US$60 million to US$380 million[12]. The HF-associated burden is expected to become an important challenge for the Chinese health system.

Depression is a common mental disorder that affects almost 3500 million people worldwide[13]. It is also common in China, with a prevalence of approximately 6.9% among Chinese adults[14], and approximately 30% of men and 40% of women aged 45 and older experience depressive symptoms[15]. Depression may increase the risk of HF through several pathways, such as decreased bone mineral density (BMD), altered bone formation and/or bone resorption, and increased risk of falls[16–18]. A recent meta-analysis of eight studies reported that depression is associated with a higher bone loss rate[16]. Falls are the major risk factor of HF, and contribute to over 95% of HF in older adults[19, 20]. And studies have shown that depression is related to higher risk of falls[17, 18], which is another potential mechanism between depression and HF.

In our previous study, we found a positive relationship between baseline depressive symptoms and HF risk in the Chinese population[21], which is in line with other studies[22–24]. However, these studies only assessed baseline depressive symptoms and failed to take into consideration that depression is a time-varying condition, the symptoms of which may relapse or remit. Most people with depression remain underdiagnosed and untreated and may suffer from fluctuating depressive symptoms[25]. Therefore, the examination of depressive symptoms as dynamic variables may provide precise estimates of the relationship between depressive symptoms and HF. However, to the best of our knowledge, no study has assessed the association between changes in depressive symptoms and the risk of HF. Thus, based on our previous study, the purposes of the current study were to further assess the association between changes in depressive symptoms and HF in middle-aged and older Chinese populations. These results may provide insights into the relationship between depressive symptoms and HF and a better understanding of whether the remission of depressive symptoms could lower the risk of HF. We hypothesized that both recent-onset and stable high depressive symptoms would be statistically related to HF risk, while remitted depressive symptoms would be nonsignificantly associated with HF.

## Methods

The current study followed the STrengthening the Reporting of Observational Studies in Epidemiology (STROBE) guidelines.

### Study population

The data were derived from the China Health and Retirement Longitudinal Study (CHARLS), a nationally representative study of individuals aged 45 and older living in mainland China. Patterned after the Health and Retirement Study (HRS) in the USA and the English Longitudinal Study of Ageing (ELSA), the CHARLS began in 2011–2012 (wave 1), and investigations were repeated every two years across 150 counties/cities and 28 provinces. Face-to-face computer-assisted personal interviews (CAPIs) were conducted in the participants’ homes by qualified interviewers. To ensure representativeness, a multilevel stratified sampling approach and probability proportional to size (PPS) sampling method were adopted. A detailed description of this project is provided elsewhere[26].

**Fig. 1:**
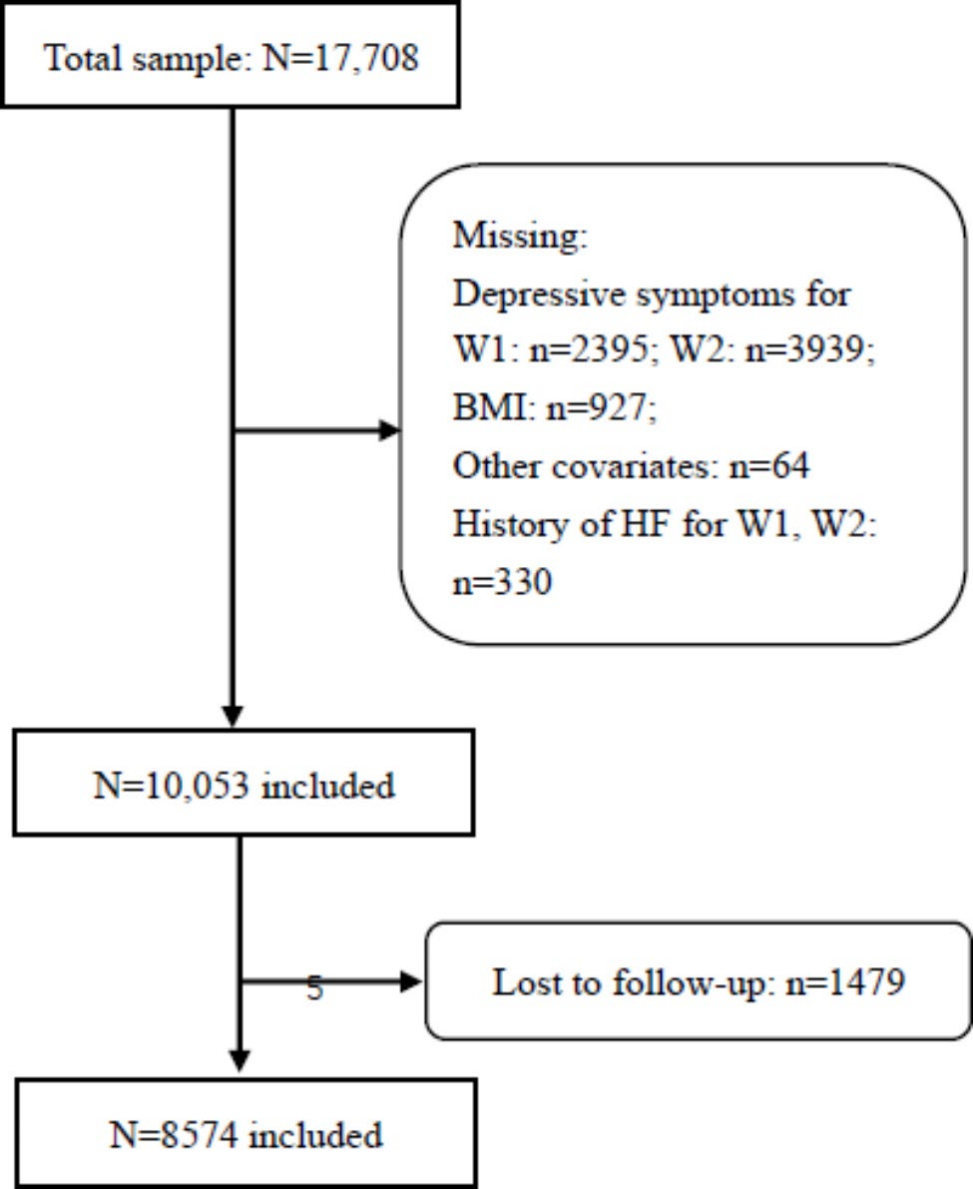
**The selection process of study participants** (W1: wave 1, the baseline survey of the China Health and Retirement Longitudinal Study (CHARLS) conducted in 2011; BMI: body mass index; HF: hip fracture; W2: wave 2, the first follow-up wave of the CHARLS conducted in 2013.)

A total of 17,708 participants were surveyed in wave 1, with wave 2 in 2013, wave 3 in 2015 and wave 4 in 2018. We excluded participants for the following reasons: a history of HF, missing data on HF, primary exposure, and other covariates. The selection process of the study participants is shown in Fig. 1. In total, 8574 individuals were included. The comparison of the baseline demographic characteristics between all participants and those included in our analyses is summarized in Supplementary Table S1.

### Depressive symptoms

Depressive symptoms were assessed using the 10-item version of the Center for Epidemiological Studied Depression scale (CESD-10), a widely used tool for the measurement of depressive symptoms in large-scale epidemiological studies[15]. The CESD-10 consists of 10 items (eight negative items and two positive items). Each item was scored as 0, 1, 2, or 3, and items 5 and 8 were reverse scored. The CESD-10 total score was calculated by summing scores across all items, ranging from 0 to 30. For each wave, a total score of 10 and above was categorized as elevated depressive symptoms. Previous studies have reported that the CESD-10 has excellent reliability and validity for older Chinese adults[15, 27].

Changes in depressive symptoms were defined based on wave 1 and wave 2, and participants were classified into four groups: (1) stable low/no: individuals without elevated depressive symptoms either at wave 1 or wave 2; (2) recent onset: individuals with new-onset depressive symptoms in wave 2, but no symptoms in wave 1; (3) recently remitted: individuals with depressive symptoms in wave 1 but not in wave 2; and (4) stable high: individuals with elevated depressive symptoms in both wave 1 and wave 2[28, 29].

### Hip fracture

The HF incidents were measured with the following two questions: “Have you ever fractured your hip?” and “Have you fractured your hip since the last interview?”. The definition of “hip bone” was also explained to each interviewee in plain words. The answer was classified as “yes” or “no”. A previous study reported good validity of self-reported HF compared with the fractures registered in the hospital register system[30].

### Covariates

The selection of covariates was based on previous studies and the availability in the CHARLS[24]. Information on baseline covariates was collected with a standardized questionnaire. Demographic characteristics included age, sex, education (below high school, high school and above), marital status (married, other [separated, widowed, divorced, never married, other]), and place of residence (rural, urban). Lifestyle-related factors included smoking status (never, current smoker, former smoker), drinking frequency (never, rarely, often). Sleep characteristics were associated with depression and HF[31, 32], and is a potential confounding factor. Therefore, sleep duration at night (< 7 h, ≥ 7 h) was also controlled. Other health-related factors included overweight or obesity (body mass index [BMI] ≥ 24.0 kg/m^2^), a history of falls, and physical comorbidities. Chronic physical diseases included hypertension, diabetes, heart problems, chronic lung diseases, liver diseases, cancers, stroke, kidney diseases, stomach or other digestive diseases, arthritis or rheumatism. Physical comorbidities were classified as 0–2 and ≥ 3 based on the number of chronic diseases.

### Statistical analysis

Descriptive statistics were used to report sample characteristics at baseline according to depressive symptom status, with analyses of variance used for comparisons between groups for continuous variables and chi-square tests used for categorical variables. To estimate the association between changes in depressive symptoms and HF incidents, three multivariable logistic regression models were fitted, with stable low/no depressive symptoms as the reference group. Model 1 was unadjusted to calculate the crude odds ratio (OR) of HF risk. Model 2 was adjusted for baseline demographic characteristics (including age, sex, education, marital status, and residence). Model 3 was further adjusted for smoking, drinking, sleep duration, a history of falls, overweight/obesity, and physical comorbidities.[22] Stratification analyses by baseline age (< 60, ≥ 60), sex, and residence were conducted to examine the associations in different subgroups. To explore the relationship between changes of CESD-10 score and HF risk, similar series of analysis were carried out. Interaction analyses were further performed through the addition of interactive terms into the multivariable models. To determine whether depressive symptoms were associated with missingness for any reason, supplementary multivariable logistic regression analyses were performed, adjusting for age and sex and using changes in depressive symptoms as explanatory variables, with missing incident HF data (yes/no) as the response variable. Finally, to test the robustness of our study, three sensitivity analyses were carried out: (1) the association between changes in depressive symptoms and HF incidents in the subsequent follow-up wave (wave 3); (2) excluding participants reporting the current intake of antidepressants, and (3) a more stringent cutoff (CESD-10 ≥ 12)[33], and repeating the analyses. All analyses were conducted using Statistical Package for the Social Sciences (SPSS), version 26.0. A two-sided p value < 0.05 was regarded as statistically significant.

## Results

A total of 8574 individuals were included in the analysis of changes in depressive symptoms and HF risk. The mean age was 58.2 years, and 51.9% were women. A total of 3075 (35.9%) participants had elevated depressive symptoms in wave 1, and compared to individuals with low depressive symptoms at baseline, those who had elevated depressive symptoms were more likely to be older, females, and have more physical comorbidities (Supplementary Table S2). There were 2637 (30.8%) participants who had a depressive symptoms rating score of 10 or greater in wave 2, indicating elevated depressive symptoms. Among all participants, 4218 (49.2%) reported low/no depressive symptoms in both wave 1 and wave 2, 1281 (14.9%) reported a recent onset of depressive symptoms, 1713 (20.0%) reported a remission of depressive symptoms and 1362 (15.9%) reported stable high depressive symptoms. The baseline characteristics of the study population across changes in depressive symptom groups are provided in Table 1. In comparison with participants with stable low depressive symptoms, those with stable high depressive symptoms were more likely to be female, less educated, unmarried, to live in rural areas, and less likely to be current smokers, to drink frequently and to have more physical comorbidities.

**Table 1:** Baseline characteristics of the China Health and Retirement Longitudinal Study (CHARLS) by depressive symptom status

Characteristics	Total n = 8574	Depressive symptom status				P value
		**stable low** **n = 4218**	**recent onset** **n = 1281**	**recent remitted n = 1713**	**stable high n = 1362**	
Age, mean(SD), y	58.2(9.1)	57.6(9.0)	58.1(9.1)	59.1(9.1)	58.9(9.1)	< 0.001
Female, n (%)	4450(51.9)	1924(45.6)	640(50.0)	1005(58.7)	881(64.7)	< 0.001
Rural residence, n (%)	5521(64.4)	2478(58.7)	854(66.7)	1180(68.9)	1009(74.1)	< 0.001
High school and above, n (%)	1086(12.7)	699(16.6)	170(13.3)	129(7.5)	88(6.5)	< 0.001
Married, n(%)	7650(89.2)	3912(92.7)	1148(89.6)	1430(83.5)	1160(85.2)	< 0.001
Smoking status, n (%)						< 0.001
Never	5205(60.7)	2442(57.9)	751(58.6)	1092(63.7)	920(67.5)	
Current smoker	2612(30.5)	1376(32.6)	412(32.2)	481(28.1)	343(25.2)	
Former smoker	757(8.8)	400(9.5)	118(9.2)	140(8.2)	99(7.3)	
Drinking frequency, n (%)						< 0.001
Never	5242(61.1)	2461(58.3)	781(61.0)	1086(63.4)	914(67.1)	
Rarely	687(8.1)	318(7.5)	116(9.1)	137(8.0)	116(8.5)	
Often	2645(30.8)	1439(34.2)	384(30.1)	490(28.6)	332(24.4)	
Overweight/obesity, n (%)	3338(38.9)	1729(41.0)	519(40.5)	614(35.8)	476(34.9)	< 0.001
Sleep duration, n (%), h						< 0.001
<7	4248(49.5)	1751(41.5)	574(44.8)	1052(61.4)	871(64.0)	
≥7	4326(50.5)	2467(58.5)	707(55.2)	661(38.6)	491(36.0)	
History of falls, n (%)	1281(14.9)	442(10.5)	159(12.4)	368(21.5)	312(22.9)	< 0.001
Comorbidities, n (%)						< 0.001
0–2	6415(74.8)	3391(80.4)	1006(78.5)	1164(68.0)	854(62.7)	
≥3	2159(25.2)	827(19.6)	275(21.5)	549(32.0)	508(37.3)	
HF, n (%)	265(3.1)	92(2.2)	55(4.3)	51(3.0)	67(4.9)	< 0.001

SD: standard deviation; HF: hip fracture. Chi-square tests were used for categorical variables, and analyses of variance were used for continuous variables.

Among individuals with no history of HF before wave 2, 265 (3.1%) reported HF incidents during the following 5-year period. In multivariable analysis (Table 2), compared with those with stable low/no depressive symptoms, participants with stable high depressive symptoms had a significantly elevated risk of HF (OR = 2.15, 95% CI, 1.53–3.02) after full adjustment for age, sex, education, marital status, residence, smoking, drinking, history of falls, sleep duration, overweight/obesity, and physical comorbidities. A recent onset of depressive symptoms was also related to an increased risk of HF (OR = 1.97, 95% CI, 1.40–2.77). No significant associations were detected among participants with remitted depressive symptoms (OR = 1.27, 95% CI, 0.89–1.82). A statistically significant association between changes of CESD-10 score and HF risk was also detected in the fully adjusted model (OR = 1.02, 95% CI, 1.00-1.04). There were no interactions between HF risk and age (p = 0.38), sex (p = 0.58) or residence (p = 0.87) in the fully adjusted models (Supplementary Table S2).

**Table 2:** Associations between changes in depressive symptoms and incident hip fracture in multivariable logistic regression analyses

Depressive symptom status	Model 1		Model 2		Model 3	
	**OR(95% CI)**	**P value**	**OR(95% CI)**	**P value**	**OR(95% CI)**	**P value**
Stable low	Ref		Ref		Ref	
Recent onset	2.01(1.43,2.83)	< 0.001	1.92(1.37,2.71)	< 0.001	1.97(1.40,2.77)	< 0.001
Recent remitted	1.38(0.97,1.95)	0.071	1.25(0.88,1.77)	0.223	1.27(0.89,1.82)	0.190
Stable high	2.32(1.68,3.20)	< 0.001	2.09(1.52,2.91)	< 0.001	2.15(1.53,3.02)	< 0.001
Changes of CESD-10 score	1.02(1.00,1.03)	0.068	1.02(1.00,1.04)	0.021	1.02(1.00,1.04)	0.014

OR: odds ratio; CI: confidence interval. Model 1 was unadjusted; Model 2 was adjusted for age, sex, residence, education and marital status;

Model 3 was further adjusted for history of falls, smoking status, drinking frequency, sleep duration, overweight/obesity, and physical comorbidities.

### Missingness analysis

In supplementary multivariable analyses (Table 3), after adjusting for age and sex, categories of changes in depressive symptoms were not associated with missingness at follow-up (all p > 0.05).

**Table 3:** Age- and sex-adjusted associations between missingness at follow-up and depressive symptoms

	Missingness at follow-up	
	**OR(95% CI)**	**P value**
Depressive symptoms status, n = 1479 missing cases		
Stable low	Ref	
Recent onset	1.09(0.93,1.28)	0.299
Recent remitted	0.97(0.84,1.13)	0.729
Stable high	0.95(0.81,1.12)	0.517

OR: odds ratio; CI: confidence interval

### Sensitivity analysis

In sensitivity analyses (Table 4), the results remained stable between changes in depressive symptoms and the risk of HF in the subsequent follow-up wave, with recent-onset (OR = 1.97, 95% CI, 1.40–2.77) and stable high (OR = 2.15, 95% CI, 1.53–3.02) depressive symptom groups having an increased risk of HF compared with the stable low/no group. Similar results were derived when we excluded participants who reported current use of antidepressants, and an alternative CESD-10 cutoff was adopted.

**Table 4:** Sensitivity analysis for the association between changes in depressive symptoms and hip fracture

	Sensitivity analysis 1		Sensitivity analysis 2		Sensitivity analysis 3	
**Depressive symptom status**	**OR(95% CI)**	**P value**	**OR(95% CI)**	**P value**	**OR(95% CI)**	**P value**
Stable low	Ref		Ref		Ref	
Recent onset	1.97(1.40,2.77)	< 0.001	1.96(1.39,2.76)	< 0.001	1.81(1.29,2.54)	< 0.001
Recent remitted	1.27(0.89,1.82)	0.190	1.25(0.87,1.80)	0.223	1.12(0.78,1.61)	0.534
Stable high	2.15(1.53.3.02)	< 0.001	2.15(1.52,3.02)	< 0.001	2.18(1.51,3.15)	< 0.001

OR: odds ratio; CI: confidence interval. Models were adjusted for age, sex, education, marital status, residence, smoking, drinking, sleep duration, fall history, overweight/obesity, and physical comorbidities. Sensitivity analysis 1: The association between changes in depressive symptoms and HF incidents in the subsequent follow-up wave (wave 3); Sensitivity analysis 2: Excluding participants reporting current use of antidepressants, and repeating the analyses; Sensitivity analysis 3: an alternative CESD-10 cutoff was used (CESD-10 ≥ 12).

## Discussion

In this nationally representative cohort study, we demonstrated that longitudinal changes in depressive symptoms were associated with HF risk. Specifically, participants with stable high- and recent-onset depressive symptoms over two consecutive biennial assessments had a 115% and 97% increased risk of HF, respectively, in comparison with those who had stable low/no symptoms. However, those who reported remitted depressive symptoms had nonsignificant differences in the risk of HF. There was no evidence of interaction effects of age, sex, or residence.

To the best of our knowledge, this is the first study to assess the relationship between changes in depressive symptoms and HF risk. Previous prospective studies have shown that individuals with elevated depressive symptoms at baseline experienced a higher risk of HF than those with low/no depressive symptoms [34, 35]. Two recent meta-analyses have examined the impact of depression or depressive symptoms on HF risk and reported pooled hazard ratios of 1.21 (95% CI = 1.11–1.31) and 1.31 (95% CI = 1.08–1.59), respectively, [22, 24]. In addition, an analysis of Taiwan’s National Health Insurance Research Database also showed that patients with depressive disorders had a 1.34-fold higher risk of HF than their counterparts[36]. However, although previous studies have observed a significant relationship between baseline depressive symptoms and HF risk, none of them have explored the impact of changes in depressive symptoms on HF. We were able to fill this gap by categorizing depressive symptoms into four groups, reflecting changes (e.g., recent-onset and recently remitted symptoms) and stable aspects (e.g., stable low/no and stable high symptoms) of depressive symptoms. Consistent with our hypothesis, we found that recent-onset and persistently high depressive symptoms were associated with an elevated risk of HF in comparison with stable low/no symptoms. Nevertheless, remitted depressive symptoms were nonsignificantly associated with HF, which suggested that the risk of HF may be reduced by effective management of depressive symptoms. Given the lack of large randomized controlled trials assessing the impact of interventions for depressive symptoms on HF risk, the evidence from large, high-quality cohort investigations is still valuable for clinical practice.

The potential mechanism of how depression affects the HF risk has not been fully elucidated but may involve two major aspects (decreased BMD and increased propensity to fall). On the one hand, depression may contribute to decreased BMD through the following pathways. First, it was reported that depression is associated with a chronic low-grade inflammatory response, and depressed patients have elevated levels of inflammatory cytokines such as interleukin-2 and interleukin-6[37], which are associated with decreased BMD[38]. Second, depression may have an impact on the concentrations of vitamin D[39], cortisol[40], and other hormones (e.g., estrogen, growth hormone)[41] that affect bone formation and/or bone resorption. Third, depression is related to unhealthy behaviors such as poor diet, alcohol consumption, cigarette smoking, physical inactivity, and poor adherence to medical recommendations that may increase the risk of osteoporosis[42–44], thus increasing the risk of HF. In addition, previous findings showed that antidepressant medications (e.g., selective serotonin reuptake inhibitors [SSRIs] and tricyclic antidepressants [TCAs]) may have a direct impact on bone metabolism and bone strength[45]. However, the use of antidepressants might be only a sign of severe depression, and interpretations of the role of antidepressants should be made with caution. Last, depression is associated with some major comorbidities, such as hypertension and diabetes[46, 47], which are potential risk factors for HF[48, 49]. Nevertheless, we controlled for these chronic physical diseases in our analyses without any significant attenuation of associations. On the other hand, for the second hypothesis, it was reported that depressed patients have a higher risk of fall accidents than their nondepressed counterparts[21, 50]. Depression may increase the risk of falls through several mechanisms. First, depression is related to neuropathological lesions in certain areas of the brain, which may affect individuals’ balance, gait, coordination, and judgment, thus increasing the risk of falls[51, 52]. Moreover, depression is also associated with decreased cognitive function and a decreased likelihood of taking necessary safety precautions, which lead to a higher fall risk[53, 54]. In addition, previous studies reported that fear of falling is associated with a higher risk of depression[55], and individuals with a higher fear of falling score fell more frequently[56–58], which may be another potential pathway.

This study has several strengths. First, this is the first study to assess the associations between changes in depressive symptoms and HF risk. With a large sample size and national representativeness, we can generalize the findings to general populations of this age. In addition, supplementary multivariable regressions and sensitivity analyses were also conducted to further complement the main results. Second, by classifying depressive symptoms into different categories, we were able to explore the effects of changes in or stable aspects of depressive symptoms on HF risk. Our findings that high- and recent-onset depressive symptoms were associated with more HF incidents than remitted symptoms provide a new direction for the prevention of HF. Furthermore, recent-onset and stable high depressive symptoms were presented in 14.9% and 15.9% of participants in this study, respectively. The corresponding population attributable risks for experiencing recent-onset and stable high depressive symptoms were 12.6% and 19.3%, respectively. These estimates may suggest that benefits of effective management of depressive symptoms can be expected in primary care. However, studies examining the effect of interventions for depressive symptoms on HF risk are scarce, and more studies are warranted on this topic.

Nevertheless, this study also has the following limitations. First, HF incidents were self-reported or proxy-reported and were impossible to confirm by medical records since those data were unavailable in the CHARLS. Although most large epidemiological surveys rely on self-report questionnaires, misclassification bias is inevitable. However, a previous study reported that self-reported HF is relatively accurate, with a positive predictive value of 94% when comparing self-reported HF with fractures registered in a hospital register system[30]. In addition, considering that HF is a serious medical condition and that patients generally seek medical examinations and treatments from physicians for HF, this limitation may not influence our conclusions. Besides, depressive symptoms were assessed with the CESD-10, a self-report tool, rather than being clinically diagnosed, which may lead to underestimations of the effects of these symptoms on HF risk. However, our analyses still showed a positive association between depressive symptoms and HF incidents, highlighting the need for early intervention of depressive symptoms in primary care among middle-aged and older Chinese individuals. In one sensitivity analysis, an alternative CESD-10 cutoff score was used, and the repeated analysis showed robustness. Second, the logistic regression method, rather than survival analysis was selected for two important reasons. on one hand, we were unable to create a time variable on the basis of the month and year for HF since these data were unavailable in the CHARLS. On the other hand, it is arbitrary and of little clinical meaning to create a time variable using the year of first reporting because the participants were interviewed biennially. Third, as we mentioned above, studies have shown that the use of antidepressants, especially SSRIs, is a potential risk factor for HF. However, information on specific antidepressant medications was unavailable in the CHARLS, limiting us to performing further analysis. However in one sensitivity analysis, we excluded those individuals who reported the use of antidepressants, and the results of the repeated analyses were stable. In addition, some confounders may change over time, yet we only controlled for confounding factors at baseline, which may cause residual bias. However, in another sensitivity analysis, we examined the associations between changes in depressive symptoms and HF incidents within a short time window, which can reduce the effect of changes in confounders on these associations, and similar results were observed. Finally, we adjusted for a wide range of possible confounders underlying the associations between depressive symptoms and HF, yet some important factors, such as dietary and physical activity, were not included because the dietary information was not collected in the CHARLS, and there was a great deal of missing information for physical activity (n = 10,678, 60.7%). However, previous studies suggested that higher physical activity is not only associated with lower HF incidence[59], but also related to less falling events[60]. And significant associations between dietary-related factors (e.g., dietary patterns, dietary diversity) and low BMD and HF were also reported[61, 62]. Therefore, residual confounding effects may still be present in current study, future investigations are needed to address this issue.

## Conclusion

In conclusion, in this large-scale study, we found that stable high and recent-onset depressive symptoms were associated with increased HF risk, while recently remitted depressive symptoms were not associated with elevated HF risk, suggesting that the HF risk may decrease if depressive symptoms remit. Our findings contribute to the limited evidence on this association. Given the rapid growth of the aging population and increasing burden of HF, interventions and support to tackle mental health (e.g., depression) are necessary in the prevention of adverse health outcomes. Future investigations conducted in other populations are warranted to extrapolate this finding to a wider population.

## Electronic supplementary material

Below is the link to the electronic supplementary material.

Supplementary Material 1. 
Table S1. The demographic characteristics of all participants and those included in our analyses. 
Table S2. Baseline characteristics of the China Health and Retirement Longitudinal Study (CHARLS) by baseline depressive symptoms. 
Table S3. Subgroup analyses for associations between changes in depressive symptoms and hip fracture risk.

## Data Availability

The data that support the findings of this study are available from the China Health and Retirement Longitudinal Study (CHARLS) group, but restrictions apply to the availability of these data, which were used under license for current study, and so are not publicly available. Data are however available from the authors upon reasonable request and with the permission of the CHARLS group (http://charls.pku.edu.cn/).

## List of abbreviations

CHARLS: China Health and Retirement Longitudinal Study.
CESD-10: 10-item version of the Center for Epidemiological Studied Depression scale.
HF: hip fracture.
OR: odds ratio.
CI: confidence interval.
BMD: bone mineral density.
HRS: Health and Retirement Study.
ELSA: English Longitudinal Study of Ageing.
CAPIs: computer-assisted personal interviews.
PPS: probability proportional to size.
BMI: body mass index.
SPSS: Statistical Package for the Social Sciences.
SSRIs: selective serotonin reuptake inhibitors.
TCAs: tricyclic antidepressants.

## References

[1] Leal J, Gray AM, Prieto-Alhambra D (2016). Impact of hip fracture on hospital care costs: a population-based study. Osteoporos international: J established as result cooperation between Eur Foundation Osteoporos Natl Osteoporos Foundation USA.

[2] Tung YC, Chang HY, Chang GM (2018). Impact of bundled payments on hip fracture outcomes: a nationwide population-based study. Int J Qual health care: J Int Soc Qual Health Care.

[3] Bliuc D, Nguyen ND, Milch VE, Nguyen TV, Eisman JA, Center JR (2009). Mortality risk associated with low-trauma osteoporotic fracture and subsequent fracture in men and women. Jama.

[4] Brauer CA, Coca-Perraillon M, Cutler DM, Rosen AB (2009). Incidence and mortality of hip fractures in the United States. Jama.

[5] Kannus P, Niemi S, Parkkari J, Palvanen M, Vuori I, Järvinen M (1999). Hip fractures in Finland between 1970 and 1997 and predictions for the future. Lancet (London England).

[6] Cooper C, Campion G, Melton LJ (1992). Hip fractures in the elderly: a world-wide projection. Osteoporos international: J established as result cooperation between Eur Foundation Osteoporos Natl Osteoporos Foundation USA.

[7] Veronese N, Maggi S (2018). Epidemiology and social costs of hip fracture. Injury.

[8] Bhandari M, Swiontkowski M (2017). Management of Acute Hip Fracture. New Engl J Med.

[9] Dhanwal DK, Dennison EM, Harvey NC, Cooper C (2011). Epidemiology of hip fracture: Worldwide geographic variation. Indian J Orthop.

[10] Cauley JA, Chalhoub D, Kassem AM, Fuleihan Gel H (2014). Geographic and ethnic disparities in osteoporotic fractures. Nat reviews Endocrinol.

[11] Fang EF, Xie C, Schenkel JA, et al. A research agenda for ageing in China in the 21st century (2nd edition): Focusing on basic and translational research, long-term care, policy and social networks. *Ageing research reviews.* 2020;64:101174.10.1016/j.arr.2020.101174. 10.1016/j.arr.2020.101174 PMC7505078 32971255

[12] Zhang C, Feng J, Wang S (2020). Incidence of and trends in hip fracture among adults in urban China: A nationwide retrospective cohort study. PLoS Med.

[13] Zhao X, Ma J, Wu S, Chi I, Bai Z (2018). Light therapy for older patients with non-seasonal depression: A systematic review and meta-analysis. J Affect disorders.

[14] Huang Y, Wang Y, Wang H (2019). Prevalence of mental disorders in China: a cross-sectional epidemiological study. The lancet Psychiatry.

[15] Lei X, Sun X, Strauss J, Zhang P, Zhao Y (2014). Depressive symptoms and SES among the mid-aged and elderly in China: evidence from the China Health and Retirement Longitudinal Study national baseline. Social Sci Med (1982).

[16] Wu Q, Liu B, Tonmoy S (2018). Depression and risk of fracture and bone loss: an updated meta-analysis of prospective studies. Osteoporos international: J established as result cooperation between Eur Foundation Osteoporos Natl Osteoporos Foundation USA.

[17] Choi NG, Marti CN, DiNitto DM, Kunik ME (2019). Longitudinal Associations of Falls and Depressive Symptoms in Older Adults. The Gerontologist.

[18] Canada B, Stephan Y, Sutin AR, Terracciano A (2020). Personality and Falls Among Older Adults: Evidence From a Longitudinal Cohort. The journals of gerontology Series B Psychological sciences and social sciences.

[19] Parkkari J, Kannus P, Palvanen M (1999). Majority of hip fractures occur as a result of a fall and impact on the greater trochanter of the femur: a prospective controlled hip fracture study with 206 consecutive patients. Calcified tissue international.

[20] Yang Y, Komisar V, Shishov N (2020). The Effect of Fall Biomechanics on Risk for Hip Fracture in Older Adults: A Cohort Study of Video-Captured Falls in Long-Term Care. J bone mineral research: official J Am Soc Bone Mineral Res.

[21] Zhu C, Yu H, Lian Z, Wang J (2022). Prospective association between depressive symptoms and hip fracture and fall among middle-aged and older Chinese individuals. BMC psychiatry.

[22] Qiu L, Yang Q, Sun N (2018). Association between depression and the risk for fracture: a meta-analysis and systematic review. BMC psychiatry.

[23] Yeh HF, Hsu YC, Clinciu DL, Tung HH, Yen YC, Kuo HC (2018). Depression and young age impact on hip fracture subsequent to stroke: A population-based cohort study. Int J Nurs Pract.

[24] Shi TT, Min M, Zhang Y, Sun CY, Liang MM, Sun YH (2019). Depression and risk of hip fracture: a systematic review and meta-analysis of cohort studies. Osteoporos international: J established as result cooperation between Eur Foundation Osteoporos Natl Osteoporos Foundation USA.

[25] Beekman AT, Geerlings SW, Deeg DJ (2002). The natural history of late-life depression: a 6-year prospective study in the community. Archives of general psychiatry.

[26] Zhao Y, Hu Y, Smith JP, Strauss J, Yang G (2014). Cohort profile: the China Health and Retirement Longitudinal Study (CHARLS). Int J Epidemiol.

[27] Chen H, Mui AC (2014). Factorial validity of the Center for Epidemiologic Studies Depression Scale short form in older population in China. Int Psychogeriatr.

[28] Gilsanz P, Kubzansky LD, Tchetgen Tchetgen EJ (2017). Changes in Depressive Symptoms and Subsequent Risk of Stroke in the Cardiovascular Health Study. Stroke.

[29] Cui Y, Zhu C, Lian Z (2021). Prospective association between depressive symptoms and stroke risk among middle-aged and older Chinese. BMC psychiatry.

[30] Hundrup YA, Høidrup S, Obel EB, Rasmussen NK (2004). The validity of self-reported fractures among Danish female nurses: comparison with fractures registered in the Danish National Hospital Register. Scandinavian J public health.

[31] Huang T, Redline S, Gordon CM, Schernhammer E, Curhan GC, Paik JM (2022). Self-reported sleep characteristics and risk for incident vertebral and hip fracture in women. Sleep health.

[32] Chunnan L, Shaomei S, Wannian L (2022). The association between sleep and depressive symptoms in US adults: data from the NHANES (2007–2014). Epidemiol psychiatric Sci.

[33] Cheng ST, Chan AC (2005). The Center for Epidemiologic Studies Depression Scale in older Chinese: thresholds for long and short forms. Int J geriatric psychiatry.

[34] Kim SY, Lee JK, Oh DJ, Kong IG, Choi HG (2019). Depression and incident hip fracture: A longitudinal follow-up study using a national sample cohort. Medicine.

[35] Mussolino ME (2005). Depression and hip fracture risk: the NHANES I epidemiologic follow-up study. Public health reports (Washington DC: 1974).

[36] Pan CC, Hu LY, Lu T, Tu MS, Shen CC, Chen ZJ (2018). Risk of hip fractures in patients with depressive disorders: A nationwide, population-based, retrospective, cohort study. PloS one.

[37] Berk M, Williams LJ, Jacka FN (2013). So depression is an inflammatory disease, but where does the inflammation come from?. BMC Med.

[38] de Pablo P, Cooper MS, Buckley CD (2012). Association between bone mineral density and C-reactive protein in a large population-based sample. Arthritis and rheumatism.

[39] de Koning EJ, Lips P, Penninx B (2019). Vitamin D supplementation for the prevention of depression and poor physical function in older persons: the D-Vitaal study, a randomized clinical trial. Am J Clin Nutr.

[40] Ganesan K, Teklehaimanot S, Tran TH, Asuncion M, Norris K (2005). Relationship of C-reactive protein and bone mineral density in community-dwelling elderly females. J Natl Med Association.

[41] Gkiatas I, Lykissas M, Kostas-Agnantis I, Korompilias A, Batistatou A, Beris A (2015). Factors affecting bone growth. Am J Orthop (Belle Mead NJ).

[42] Alghadir AH, Gabr SA, Al-Eisa E (2015). Physical activity and lifestyle effects on bone mineral density among young adults: sociodemographic and biochemical analysis. J Phys therapy Sci.

[43] Cashman KD (2007). Diet, nutrition, and bone health. J Nutr.

[44] Grenard JL, Munjas BA, Adams JL (2011). Depression and medication adherence in the treatment of chronic diseases in the United States: a meta-analysis. J Gen Intern Med.

[45] Rabenda V, Nicolet D, Beaudart C, Bruyère O, Reginster JY (2013). Relationship between use of antidepressants and risk of fractures: a meta-analysis. Osteoporos international: J established as result cooperation between Eur Foundation Osteoporos Natl Osteoporos Foundation USA.

[46] Ojike N, Sowers JR, Seixas A (2016). Psychological Distress and Hypertension: Results from the National Health Interview Survey for 2004–2013. Cardiorenal Med.

[47] Wesołowska K, Elovainio M, Hintsa T (2018). Is the association between depressive symptoms and glucose bidirectional? A population-based study. Health psychology: official journal of the Division of Health Psychology American Psychological Association.

[48] Li C, Zeng Y, Tao L, et al. Meta-analysis of hypertension and osteoporotic fracture risk in women and men. *Osteoporosis international: a journal established as result of cooperation between the European Foundation for Osteoporosis and the National Osteoporosis Foundation of the USA.* 2017;28(8):2309–2318.10.1007/s00198-017-4050-z. 10.1007/s00198-017-4050-z 28447105

[49] Bai J, Gao Q, Wang C, Dai J (2020). Diabetes mellitus and risk of low-energy fracture: a meta-analysis. Aging Clin experimental Res.

[50] Kvelde T, Lord SR, Close JC (2015). Depressive symptoms increase fall risk in older people, independent of antidepressant use, and reduced executive and physical functioning. Archives of gerontology and geriatrics.

[51] Bouckaert F, De Winter FL, Emsell L (2016). Grey matter volume increase following electroconvulsive therapy in patients with late life depression: a longitudinal MRI study. J psychiatry neuroscience: JPN.

[52] Fossati P, Radtchenko A, Boyer P (2004). Neuroplasticity: from MRI to depressive symptoms. Eur neuropsychopharmacology: J Eur Coll Neuropsychopharmacol.

[53] Richmond RL, Law J, Kay-Lambkin F (2011). Physical, mental, and cognitive function in a convenience sample of centenarians in Australia. J Am Geriatr Soc.

[54] Anstey KJ, Burns R, von Sanden C, Luszcz MA (2008). Psychological well-being is an independent predictor of falling in an 8-year follow-up of older adults. The journals of gerontology Series B Psychological sciences and social sciences.

[55] Yao Q, Jin W, Li Y (2021). Associations between fear of falling and activity restriction and late life depression in the elderly population: Findings from the Irish longitudinal study on ageing (TILDA). J Psychosom Res.

[56] Scholz M, Haase R, Trentzsch K, Weidemann ML, Ziemssen T (2021). Fear of falling and falls in people with multiple sclerosis: A literature review. Multiple Scler Relat disorders.

[57] Lavedán A, Viladrosa M, Jürschik P (2018). Fear of falling in community-dwelling older adults: A cause of falls, a consequence, or both?. PloS one.

[58] Mak MK, Pang MY (2009). Fear of falling is independently associated with recurrent falls in patients with Parkinson’s disease: a 1-year prospective study. J Neurol.

[59] LaMonte MJ, Wactawski-Wende J, Larson JC (2019). Association of Physical Activity and Fracture Risk Among Postmenopausal Women. JAMA Netw open.

[60] Kendrick D, Kumar A, Carpenter H (2014). Exercise for reducing fear of falling in older people living in the community. The Cochrane database of systematic reviews.

[61] Fabiani R, Naldini G, Chiavarini M (2019). Dietary Patterns in Relation to Low Bone Mineral Density and Fracture Risk: A Systematic Review and Meta-Analysis. Adv Nutr (Bethesda Md).

[62] Zhang J, Liang D, Zhao A. Dietary Diversity and the Risk of Fracture in Adults: A Prospective Study. Nutrients 2020;12(12)10.3390/nu12123655. 10.3390/nu12123655 PMC7761242 33261013

