# Supplementary material for: Association between changes in depressive symptoms and hip fracture among middle-aged and older Chinese individuals: a prospective cohort study
Source: BMC Geriatr. 2022 Nov 8;22:844. doi: 10.1186/s12877-022-03484-8 (PMC9644634; doi:10.1186/s12877-022-03484-8)
Supplement: Supplementary file 1 — Supplementary Material 1. Table S1. The demographic characteristics of all participants and those included in our analyses. Table S2. Baseline characteristics of the China Health and Retirement Longitudinal Study (CHARLS) by baseline depressive symptoms. Table S3. Subgroup analyses for associations between changes in depressive symptoms and hip fracture risk. [file 12877_2022_3484_MOESM1_ESM.docx]

**Supplementary materials**

Table S1. The demographic characteristics of all participants and those included in our analyses.

| Characteristics | All participants (n=17708) | Those included in our analysis  (n=8574) |
| --- | --- | --- |
| Age, mean | 59.2 | 58.2 |
| Sex (%) |  |  |
| Male | 48.0 | 48.1 |
| Female | 52.0 | 51.9 |
| Place of residence (%) |  |  |
| Rural | 59.5 | 64.4 |
| Urban | 40.5 | 35.6 |
| Marital status (%) |  |  |
| Married | 87.3 | 89.2 |
| Others | 12.7 | 10.8 |
| Education (%) |  |  |
| Below high school | 87.2 | 87.3 |
| High school and above | 12.8 | 12.7 |

HF: hip fracture.

Table S2. Baseline characteristics of the China Health and Retirement Longitudinal Study (CHARLS) by baseline depressive symptoms

| Characteristics | Total n=8574 | Elevated depressive symptoms at baseline | | P value |
| --- | --- | --- | --- | --- |
|  |  | No, n=5499 | Yes, n=3075 |  |
| Age, mean(SD), y | 58.2(9.1) | 57.7(9.1) | 59.0(9.1) | <0.001 |
| Female, n(%) | 4450(51.9) | 2564(46.6) | 1886(61.3) | <0.001 |
| Rural residence, n(%) | 5521(64.4) | 3332(60.6) | 2189(71.2) | <0.001 |
| High school and above, n(%) | 1086(12.7) | 869(15.8) | 217(7.1) | <0.001 |
| Married, n(%) | 7650(89.2) | 5060(92.0) | 2590(84.2) | <0.001 |
| Smoking status, n(%) |  |  |  | <0.001 |
| Never | 5205(60.7) | 3193(58.1) | 2012(65.4) |  |
| Current smoker | 2612(30.5) | 1788(32.5) | 824(26.8) |  |
| Former smoker | 757(8.8) | 518(9.4) | 239(7.8) |  |
| Drinking frequency, n(%) |  |  |  | <0.001 |
| Never | 5242(61.1) | 3242(59.0) | 2000(65.0) |  |
| Rarely | 687(8.1) | 434(7.9) | 253(8.2) |  |
| Often | 2645(30.8) | 1823(33.2) | 822(26.7) |  |
| Overweight/obesity, n(%) | 3338(38.9) | 2248(40.9) | 1090(35.4) | <0.001 |
| Sleep duration, n(%), h |  |  |  | <0.001 |
| <7 | 4248(49.5) | 2325(42.3) | 1923(62.5) |  |
| ≥7 | 4326(50.5) | 3174(57.7) | 1152(37.5) |  |
| History of fall, n(%) | 1281(14.9) | 601(10.9) | 680(22.1) | <0.001 |
| Comorbidities, n(%) |  |  |  | <0.001 |
| 0-2 | 6415(74.8) | 4397(80.0) | 2018(65.6) |  |
| ≥3 | 2159(25.2) | 1102(20.0) | 1057(34.4) |  |

SD: standard deviation. Chi-square tests were used for categorical variables, and t-tests were used for continuous variables.

Table S3. Subgroup analyses for associations between changes in depressive symptoms and hip fracture risk

|  | Stable low | | Recent-onset | | Recent remitted | | Stable high | | P-interaction |
| --- | --- | --- | --- | --- | --- | --- | --- | --- | --- |
|  | OR(95%CI) | P value | OR(95%CI) | P value | OR(95%CI) | P value | OR(95%CI) | P value |  |
| Age |  |  |  |  |  |  |  |  | 0.381 |
| 45-60 | Ref |  | 2.10(1.35,3.27) | 0.001 | 1.12(0.68,1.86) | 0.658 | 1.55(0.95,2.55) | 0.082 |  |
| ≥60 | Ref |  | 1.74(1.01,2.99) | 0.047 | 1.45(0.86,2.42) | 0.160 | 2.85(1.76,4.60) | <0.001 |  |
| Sex |  |  |  |  |  |  |  |  | 0.575 |
| Men | Ref |  | 1.58(0.97,2.59) | 0.069 | 1.35(0.81,2.25) | 0.250 | 2.15(1.30,3.56) | 0.003 |  |
| Women | Ref |  | 2.42(1.49,3.94) | <0.001 | 1.23(0.74,2.04) | 0.433 | 2.16(1.35,3.47) | 0.001 |  |
| Residency |  |  |  |  |  |  |  |  | 0.873 |
| Rural | Ref |  | 2.02(1.35,3.02) | 0.001 | 1.18(0.77,1.81) | 0.444 | 2.02(1.36,3.00) | 0.001 |  |
| Urban | Ref |  | 1.78(0.92,3.45) | 0.089 | 1.54(0.80,2.99) | 0.199 | 2.50(1.30,4.84) | 0.006 |  |

OR: odds ratio; CI: confidence interval. Models were adjusted for age, sex, education, marital status, residence, smoking, drinking, sleep duration, fall history, overweight/obese, physical comorbidities, as appropriate.
